# Mapping the Celebrity Endorsement of Branded Food and Beverage Products and Marketing Campaigns in the United States, 1990–2017

**DOI:** 10.3390/ijerph16193743

**Published:** 2019-10-04

**Authors:** Mi Zhou, Srijith Rajamohan, Valisa Hedrick, Sofia Rincón-Gallardo Patiño, Faiz Abidi, Nicholas Polys, Vivica Kraak

**Affiliations:** 1 Department of Human Nutrition, Foods, and Exercise, Virginia Tech, Blacksburg, VA 24061, USA; 2 Advanced Research Computing, Virginia Tech, Blacksburg, VA 24061; USA

**Keywords:** celebrity endorsement, food and beverage products, Smart Snacks Standards, United States

## Abstract

Celebrity endorsement used to promote energy-dense and nutrient-poor (EDNP) food and beverage products may contribute to poor dietary habits. This study examined celebrity endorsement of branded food and beverage products and marketing campaigns in the United States (US) from 1990 to 2017. Celebrity endorsement data were collected from peer-reviewed and grey literature. Interactive data visualizations were created for the endorsement relationships between celebrities, companies and products whose nutritional profiles were compared with the US Department of Agriculture’s (USDA’s) Smart Snacks Standards. Logistic regression was used to explore associations between celebrities’ demographic profiles and the nutritional profiles of products. Results showed 542 celebrities were associated with 732 endorsements representing 120 brands of 59 companies across 10 food and beverage categories. Two thirds (67.2%; *n* = 80) of the brands represented EDNP products that did not align with the USDA’s Smart Snacks Standards. Logistic regression analysis indicated that Millennial (*p* = 0.008) and male celebrities (*p* = 0.041) were more likely to endorse EDNP products than Generation Z teen and female celebrities, respectively. No statistical significance was observed for celebrities of other demographic profiles. This study may inform future policies and actions of the US government, industry, researchers and consumer advocacy organizations to use celebrity endorsement to promote healthy food environments for Americans.

## 1. Introduction

Overweight and obesity are public health threats in the United States (US) that affect more than two-thirds (70.2%) of adults, aged 20 years and older, and nearly one-third (32.4%) of children and adolescents, aged 2–19 years [1,2]. The marketing of energy-dense and nutrient-poor (EDNP) food and beverage products by food, beverage and restaurant companies has resulted in excessive intake of EDNP products including sugar-sweetened beverages (SSBs), candy, and quick service restaurant (QSR) meals that have contributed to poor diet quality and rising obesity rates among Americans [3,4,5,6,7,8].

The 2015–2020 Dietary Guidelines for Americans (DGA) encourage individuals to eat a nutrient-dense healthy diet to maintain a healthy weight and reduce the prevalence of obesity and diet-related non-communicable diseases [9]. Most Americans do not consume a high quality and diverse diet aligned with the DGA measured by the United States Department of Agriculture’s (USDA’s) Healthy Eating Index score, which increased only slightly from 56 in 2005–2006 to 59 out of 100 in 2013–2014 [10].

The Healthy Hunger-Free Kids Act of 2010 is a public health law that substantially strengthened the nutrition standards used to guide the meals and snacks served to American children and adolescents in school settings from grades K–12 [11]. The USDA adopted new nutrition standards for school meals in 2012–2013 and school snacks in 2014 that aligned with the DGA for all foods and beverages served in school settings [12,13]. The Healthy Hunger-Free Kids Act of 2016 rules require local education agencies to include wellness policies to prohibit the marketing of food and beverage products that are not consistent with the USDA’s Smart Snacks in School nutrition standards on school properties during the school day [14,15]. The USDA nutrition standards are the best available to assess the types of food and beverage products that should be marketed to children and adolescents both within and outside school settings, and support the development of life-long healthy eating patterns through adulthood [12,13].

Many integrated marketing communications strategies are used to promote EDNP products to children, adolescents and young adults, which increases their risk of obesity [16]. Celebrity endorsement is one of these marketing strategies that represents a person who uses his/her public recognition to promote the sales, use or consumption of a brand, product or service [17]. Marketing research describes various theoretical or conceptual models such as the source credibility model [18] and product match-up hypothesis [19] to explain the influence of celebrity endorsement on people’s decisions and behaviors. Businesses engage with celebrities through partnerships and sponsorships to establish commercially beneficial relationships. Successful partnerships are based on the celebrities’ credibility (measured by trust, attractiveness and expertise) and congruence (represented by the fit between a celebrity and a target group, and between the celebrity and a brand or product line). These partnerships generate brand recognition and revenue for companies and celebrities [18,19].

Celebrity endorsement creates positive brand attitudes, encourages purchase intentions and enhances brand loyalty among targeted consumers [20,21,22]. Experimental studies conducted in Australia and the United Kingdom (UK) have suggested that both children and their parents are more likely to consume EDNP products after viewing advertisements that feature celebrities [23,24,25]. In the US, celebrity endorsement increased from 15% to 25% of all advertisements between 1979 and 1997 [26,27]. The Federal Trade Commission’s (FTC’s) 2012 report on food marketing to children and adolescents documented the expenditures of 48 food, beverage, restaurant and entertainment companies. The report stated that the celebrity endorsement expenditures of child- and adolescent-directed food and beverage marketing increased from $26.8 million in 2006 to $99.3 million in 2009 mainly for EDNP products [28]. Descriptive studies conducted in the US concluded that most foods and beverages endorsed by sports and music celebrities targeted to children and adolescents are EDNP products [29,30], and the positive images of athletes are particularly used by food, beverage and restaurant companies to promote EDNP products to youth [31].

US marketing research suggests that Generation Z teens (born 1995–2010) and Millennials (born 1981–1994), men or Black or African Americans are more likely to be influenced by celebrities with whom they share similar demographic characteristics [19,27,32,33,34,35,36]. Companies use targeted marketing as a strategy to align celebrities who share similar demographic profiles, which may raise ethical concerns for racially and ethnically diverse young people [37].

Previous studies [29,30] have not included celebrities other than professional athletes and musicians, or those involved in marketing campaigns that encouraged healthy dietary habits such as eating fruits and vegetables or consuming milk or water. Additionally, the nutrition evaluation of products in previous studies used the UK’s nutrient profile model that differs from the DGA and the USDA nutrition standards. Furthermore, no published study has comprehensively explored the relationships among celebrities’ demographic profiles (i.e., sex, age, race/ethnicity and profession); their associations with the companies or non-governmental organizations’ (NGOs’) health-promotion and marketing campaigns; and the nutritional profiles of the endorsed food and beverage products in the US marketplace. This study addresses this research gap.

### Study Purpose

This study explored celebrity endorsement used by US food, beverage and restaurant firms and NGOs that promoted branded food and beverage products, and branded health or marketing campaigns to Americans between 1990 and 2017. We anticipate that the results of this study could inform future policies and actions of the US government, industry and consumer advocacy organizations to use celebrity endorsements to promote healthy food environments for Americans.

## 2. Materials and Methods

### 2.1. Research Questions and Hypotheses

This study was guided by three research questions (RQs) listed below.

RQ1: What food and beverage products or brands did celebrities endorse in the US between 1990 and 2017?
RQ2: What proportion of products endorsed by US celebrities do not align with government-recommended nutrition guidelines?
RQ3: What are the associations between the US celebrities’ demographic profiles (i.e., profession, sex, age, and race/ethnicity) and the nutritional profiles of the endorsed food and beverage products or brands?

The first research question used descriptive statistics, so no hypothesis was established. For RQ2, we hypothesized that celebrities were more likely to endorse EDNP food and beverage products that do not align with the DGA and the USDA’s Smart Snacks Standards than products that align with these healthy dietary guidelines. For RQ3, we hypothesized that celebrities are more likely to endorse EDNP food and beverage products if they are Black or African American, male, athletes, Generation Z teens or Millennials, compared to celebrities of other demographic profiles.

First, we created a database that organized, analyzed and visually mapped celebrity endorsements used by US food, beverage, restaurant firms and NGOs between 1990 and 2017. Second, we analyzed the nutritional profiles of the endorsed food and beverage products and brands based on their alignments with the USDA’s Smart Snacks Standards. Third, we used logistic regression to examine the associations between the celebrity demographic profiles and the nutritional profiles of the endorsed food and beverage products or brands.

### 2.2. Search Strategy and Data Collection

A sample was collected of celebrity endorsements for food and beverage products between 1 January 1990 and 31 December 2017 through a comprehensive review of evidence that included: (1) four electronic scientific databases (i.e., PubMed, Business Source Complete, PsycINFO and Google Scholar); (2) grey-literature sources (i.e., books, reports or briefs published by government, academic institutions, private foundations, industries or industry associations); (3) Google search engine of relevant websites (i.e., government agencies, NGOs, food, beverage and restaurant companies, and food retailers); and (4) media sources (i.e., social media, news and press releases). We used 1990 as the start of the search date because the Got Milk? and Milk Mustache Campaigns were initiated during the mid-1990s by the Milk Processor Education Program (MilkPEP) to promote fluid milk and were associated with many US celebrities over the past 20 years [38].

The search terms included “celebrity” or “athlete” or “entertain” and “endorsement” or “advertising” or “marketing” and “food” or “beverage” or “drink”. The inclusion criteria were living human celebrities who are currently or have been associated with one or more endorsements of US food, beverage and restaurant brands or products or branded US health promotion or marketing campaigns. The campaigns in this study included: Got Milk? and Milk Mustache Campaigns [38], the Partnership for a Healthier America’s (PHA’s) Drink Up Campaign to promote water [39] and Fruits and Veggies (FNV) Campaign to promote fruits and vegetables [40]. The exclusion criteria were: (1) celebrity endorsements not in the US marketplace; (2) non-human celebrities and cartoon characters; (3) celebrity endorsements used to promote tobacco, alcohol, dietary supplements, diet products and medications; and (4) food and beverage or campaign endorsements before 1990. Nutrition information was collected for food and beverage products from the company websites between May and December 2018. If the nutrition information was not available online, product labels were checked in a major retail grocery store located in Blacksburg, Virginia between May and December 2018.

### 2.3. Data Coding

#### 2.3.1. Coding Celebrity Demographic Information

Each search result for a celebrity name and his/her demographic and related endorsement information was entered into an Excel database between 1 January 2016 and 31 December 2017. Celebrities were classified into three categories based on their profession including entertainers (i.e., actors, actresses, dancers, film directors, illusionists, models, musicians and television personalities); professional athletes (i.e., ball players, bikers, boxers, car racers, ice sports athletes, wrestlers, muscle artists, track and field athletes, surfers, swimmers and gymnasts); and others (i.e., celebrity chefs, politicians and business entrepreneurs). Celebrities’ demographic information was coded based on the Internet search and grey-literature sources for sex (i.e., male/female); marketing age segmentation (i.e., Generation Z teens born 1995–2012, Millennials born 1981–1994, Generation X born 1966–1980, Baby Boomers born 1961–1965, and the Silent Generation born 1950 or earlier) [41]; and race/ethnicity (i.e., White, Black, Latino(a), Asian and Multi-racial). Each celebrity entry in the Excel database was associated with a weblink that verified a visual or descriptive association between the celebrity and the companies, organizations, brands and product endorsements.

#### 2.3.2. Coding Food and Beverage Categories

Table 1 summarizes the 10 food and beverage categories used to code the endorsements in the Excel database, which were adapted from several US nutrition standards. The FTC’s 2012 report of food marketing to children and adolescents classified food and beverage products into 10 categories that included: QSR foods, carbonated beverages, breakfast cereals, snack foods, juice and non-carbonated beverages, candy or frozen desserts, dairy products, prepared foods and meals, baked foods, fruits and vegetables [28]. The Healthy Eating Research (HER) 2013 recommendations for healthier beverages and the USDA’s Smart Snacks Standards further divided the beverage categories into SSBs (more than 60 calories per 12 fluid ounces), low-calorie beverages (LCBs) (60 or less calories per 12 fluid ounces), no-calorie beverages (NCBs) and water [13,42].

#### 2.3.3. Coding Food and Beverage Products’ Nutritional Profiles

The 2015–2020 DGA emphasize healthy eating patterns instead of setting nutrition standards for the intake of specific foods or nutrients [9]. Therefore, the USDA’s Smart Snacks Standards were used to evaluate the proportion of products that met the government-recommended nutritional profiles [13]. The Smart Snacks Standards have encouraged companies to sell products including entrées, side dishes, snacks, and beverages that offer whole grains, fruits, vegetables, low-fat or non-fat dairy or protein as the first ingredient; combine foods that contain at least a quarter cup of fruits and/or vegetables; and meet specific nutrient targets for the amount and percentage of calories, fats (i.e., saturated and *trans* fats), sodium and sugars per serving (Table 2) [13].

The food and beverage endorsements of the celebrities were classified into three groups. Group one included celebrities who endorsed specific food or beverage products (i.e., Coca-Cola, Pepsi, Fruity Fruits) that contained specific nutritional profile information. Group two included celebrities who endorsed a corporate or company brand (e.g., McDonald’s, Kemps or Borden) that contained products of various nutritional profiles. Group three included celebrities who endorsed a social marketing or health-promotion campaign brand.

The nutrition information (i.e., fat, saturated fat, *trans* fat, sugar, sodium, serving size) for each group one and group two product was entered into the Smart Snacks Product Calculator established by the Alliance for a Healthier Generation to assist the US school staff to ensure foods and beverages align with the USDA’s Smart Snacks Standards [43]. The standards alignments for group one products were decided based on the results generated by the Smart Snacks Product Calculator. Group two brands that contained more than 50% of products that met the Smart Snacks Standards were classified as aligning with these standards. For group three, the Drink Up and the FNV Campaigns were classified as aligning with the Smart Snacks Standards because all celebrities involved in these campaigns promoted only the generic products associated with the campaign brands that aligned with the Smart Snacks Standards [39,40]. However, celebrities associated with the Got Milk? and Milk Mustache Campaigns promoted a range of dairy products, including skim milk and 1% milk that aligned with the Smart Snacks Standards, and whole milk and chocolate milk that did not align with the USDA’s Smart Snacks Standards [38]. We were unable to differentiate which celebrities were associated with certain dairy products only based on the endorsement photographs. Therefore, we included the celebrity endorsements of the Got Milk? and Milk Mustache Campaigns to answer RQ1 but excluded these data to answer RQ2 and RQ3.

To increase the scientific rigor of the coding process, the data were entered by the primary researcher (M.Z.) and checked by two data scientists (S.R. and F.A.). The data coding process was verified by co-investigators who had expertise in food, nutrition, public health and marketing research (V.H., V.K. and S.R.-G.P.). The coding discrepancies were further checked from the original resources and consensus was obtained across all co-investigators.

### 2.4. Data Analyses

To answer RQ1, the frequencies of unique celebrities, food and beverage companies, brands, categories, total endorsements and celebrity demographic characteristics were calculated using SPSS statistical software version 24 for Windows (IBM Corp., Armonk, NY, USA, 2016) [44]. We also created interactive tree diagrams or dendrograms to illustrate the endorsement relationships between the celebrities, companies or organizations, and the products or brands. The data for the dendrograms were generated using the Python programming language version 3.5.5 (Python Software Foundation, Wilmington, DE, USA, 2018) [45]. The resulting data were visualized using the JavaScript D3 library version 5.7.0 (GitHub Inc., San Francisco, CA, USA, 2018) [46]. The dendrograms are compatible with most web browsers, ensuring universal open access.

To answer RQ2, a Chi-squared goodness of fit test was conducted using SPSS to explore whether there was a statistically significant difference (*p* < 0.05) between the proportion of endorsements, products or brands that aligned with the USDA’s Smart Snacks Standards and those did not align with these standards.

To answer RQ3, we used a series of Chi-squared of independence tests to check if there were any statistically significant associations between the variables in each demographic category. A conditional logistic regression model was then built to describe, explain and predict the relationships between the dependent binary variables (i.e., align or did not align with the Smart Snacks Standards) and all the independent variables (endorsements associated with the celebrities’ demographic profiles). The independent variables stated in the hypothesis were set up as the reference variables. Other independent variables in each demographic group were compared with the reference variables. The odds ratio of each comparison generated by the logistic regression model was used to quantify how likely these endorsements were associated with products or brands that aligned with the Smart Snacks Standards. The validity of this model was evaluated using the Hosmer–Lemeshow goodness of fit test and the model’s predicted probability. SPSS was used for the data analyses, and the significance level was set at *p* < 0.05.

## 3. Results

### 3.1. RQ1: Celebrity Endorsements of Food and Beverage Products in the US between 1990 and 2017

The data analysis revealed that 542 celebrities were associated with 732 endorsements representing 120 products or brands of 59 companies or organizations across 10 food and beverage categories. Table 3 describes the results that entertainment, male, white, and Millennial celebrities were the most common in their respective demographic categories.

**Figure 1:**
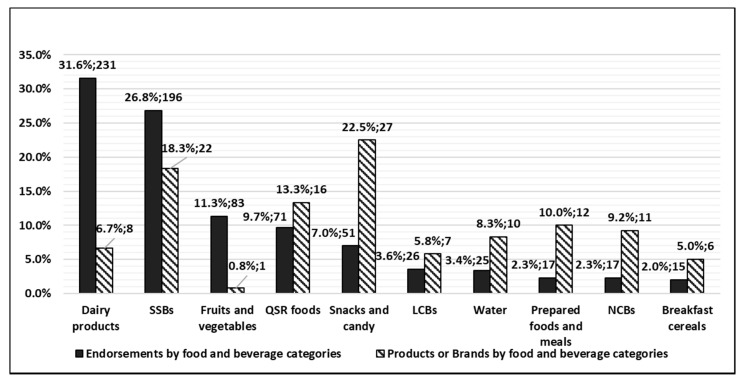
Comparison for the percentages and numbers of endorsements (*n* = 732) and the number of unique brands or products (*n* = 120) by food and beverage categories (*n* = 10). Abbreviations: quick-service restaurant (QSR); sugar-sweetened beverage (SSB); low-calorie beverage (LCB); no-calorie beverage (NCB).

Due to the multiple endorsement relationships between celebrities and the food and beverage brands or products, the number of endorsements was greater than either the number of unique celebrities or the number of unique brands or products. Figure 1 shows the comparison between the 732 endorsements and the 120 unique brands or products according to 10 food and beverage categories. According to the analysis based on the 732 endorsements, the top three food and beverage categories were represented by dairy, SSB, and fruit and vegetable brands or products. However, when analyzed by the 120 unique products or brands, the distribution was different and the top three food and beverage categories were associated with snacks and candy, SSB and QSR products or brands. Dairy products or brands ranked first by the number of endorsements but ranked eighth by the number of products and brands. Snacks and candy ranked fifth when calculated by the number of endorsements, but ranked first according to the number of products and brands. SSBs ranked second for endorsements and brands.

**Figure 2:**
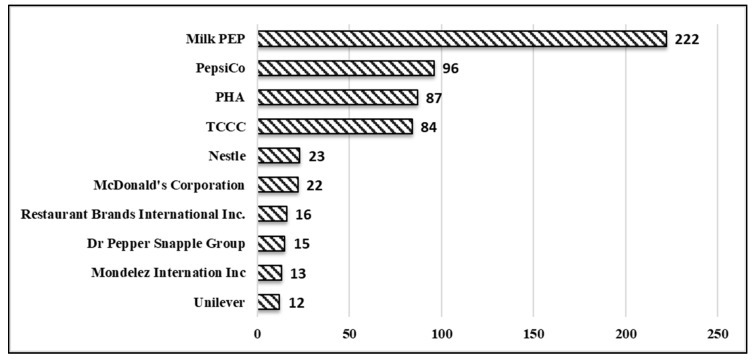
Top 10 companies or organizations ranked by number of partnered celebrities to promote food and beverage products or brands. Abbreviations: Milk Processor’s Education Program (MilkPEP); Partnership for a Healthier America (PHA); The Coca-Cola Company (TCCC).

Figure 2 summarizes the top 10 companies or NGOs (i.e., MilkPEP or PHA) according to the number of partnered celebrities. Of the 59 companies or organizations, the MilkPEP ranked as the top organization with 222 celebrities involved in the Got Milk? and Milk Mustache Campaigns that ran for 20 years from 1995 to 2015. PepsiCo ranked second with 96 celebrities who endorsed mostly EDNP food and beverage products, especially salty snacks and SSB products and brands. A few other food and beverage firms including The Coca-Cola Company (TCCC), Mondelez International and McDonald’s Corporation were highly ranked with celebrity endorsements that featured primarily EDNP products.

As a single product or brand could use multiple celebrity endorsers in advertisements to appeal to different types of consumers, and vice versa, one celebrity could endorse multiple brands or products. Our results showed that more than half (53.7%; *n* = 65) of the products or corporate brands hired only one celebrity; more than one third (38.8%; *n* = 47) partnered with two to nine celebrities; and less than one-tenth (7.4%; *n* = 9) were associated with more than 10 celebrities. Results also demonstrated that of the 542 celebrities, more than three quarters (79.2%; *n* = 429) endorsed only one product or brand; less than a fifth (19.6%; *n* = 106) endorsed two to four products or brands; and only 1.3% (*n* = 7) endorsed five or more products or brands.

Table 4 describes the frequencies and percentages of endorsements (*n* = 732) for each food and beverage category based on the celebrity demographic profiles. The greatest percentage of the endorsements for black, male, sports, and Millennial celebrities represented SSB brands or products. Most endorsements for white, Latino(a), Asian, female, entertainment, Generation X, Baby Boomer and Silent Generation celebrities were dairy brands or products. Multi-racial and Generation Z celebrities were mostly associated with fruit and vegetable brands (i.e., FNV) or products; and celebrities in the other profession category were mostly involved in prepared food and meal endorsements.

**Figure 3:**
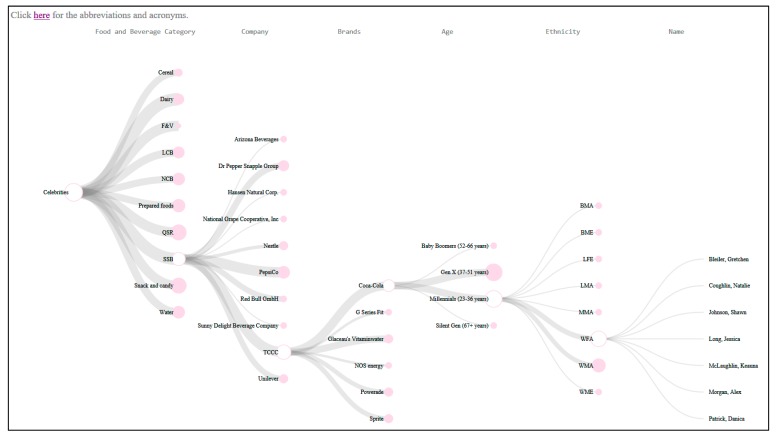
Screenshot of an interactive dendrogram showing the celebrity endorsement relationships by the food and beverage category (*n* = 10), companies (*n* = 59) and celebrities (*n* = 542). We created two dendrograms that emphasized the endorsement relationships from different perspectives: dendrogram one emphasizes the food and beverage category endorsements and dendrogram two emphasizes the company endorsements. The two interactive dendrograms can be viewed online [47] in any web browser by disabling the browser protection to enable the loading of files required for the D3-based visualization.

Figure 3 provides the screenshot of an interactive dendrogram that describes the endorsement relationships between celebrities and food/beverage categories, and between celebrities and food/beverage companies or organizations. Each solid node can be expanded to obtain more detailed information, and the size difference of the nodes represents the different amounts of the expanded items. Abbreviations are explained by clicking the “here” link. The dendrogram can be viewed interactively through an online web browser where readers can select various solid nodes to see the different endorsement relationships for each food and beverage category (*n* = 10), each company (*n* = 59) and each celebrity (*n* = 542).

### 3.2. RQ2: Comparison between EDNP and Healthy Product Endorsements

Table 5 summarizes the nutritional profile analyses for the food and beverage product endorsements based on the Smart Snacks Standards Calculator. This table indicates that among the 510 endorsements (excluding Got Milk? and Milk Mustache Campaigns), two-thirds of the represented food and beverage products or brands did not align with the USDA’s Smart Snacks Standards. Similarly, among the 119 unique food and beverage products or brands (excluding the Got Milk? and Milk Mustache Campaigns), the brands associated with food and beverage products that did not align with the USDA’s standards were two times greater than those aligned with the standards. Furthermore, two-thirds of the 356 celebrities (excluding the celebrities involved in the Got Milk? and Milk Mustache Campaigns) associated with food and beverage products did not align with the Smart Snacks Standards. A total of 7% (*n* = 38) of celebrities endorsed products both aligned and not aligned with the USDA standards.

### 3.3. RQ3: Associations between Celebrity Demographic Profiles and Nutritional Profiles of Endorsed Food and Beverage Products or Brands

Table 6 shows the results of the Chi-squared and the logistic regression analyses of the variables (excluding the endorsements of the Got Milk? and Milk Mustache Campaigns). The Chi-squared analyses indicated that sex and age of celebrities were significantly associated with the nutritional profiles of their endorsed food and beverage products, while the profession and race/ethnicity of celebrities were not significantly associated with the nutritional profiles of their endorsed food and beverage products. The logistic regression model shows that male celebrities were associated with 1.5 times more endorsements for EDNP products that did not align with the USDA’s Smart Snacks Standards compared with female celebrities (*p* < 0.05). Millennial celebrities (aged 23–36 years and born 1981–1994) were seven times more likely to be associated with EDNP products than Generation Z celebrities (aged 17–22 years and born 1995–2010) (*p* < 0.05). This conditional logistic regression model was a good fit for the variables (*p* = 0.127) according to the Hosmer–Lemeshow goodness of fit test, with demographic profiles of celebrities accounting for 68.4% of the variance associated with endorsing EDNP food and beverage products.

## 4. Discussion

### 4.1. Research Findings and Implications for Policies

This is the first US study to offer a comprehensive examination of the relationships among celebrities, food and beverage products or brands, and various companies or NGOs that used celebrity endorsements in health-promotion or marketing campaigns. It is also the first study to use the USDA’s Smart Snacks Standards to analyze the nutritional profiles of the food and beverage products or brands endorsed by celebrities in the US marketplace over more than 20 years.

The great number of celebrities involved in the Got Milk? and Milk Mustache Campaigns and the FNV Campaign elevated the number of endorsements for dairy, fruit and vegetable promotion, which might misrepresent the nature of celebrity endorsement used for food and beverage marketing in the US. However, the number of unique brands or products showed that a great majority of the endorsements were for brands associated with SSB, QSR, snacks and candy products rather than for dairy products, fruits and vegetables.

The two interactive dendrograms provide a panorama view of how celebrity endorsements have been used to promote branded food and beverage products and branded health and marketing campaigns in the US from 1990 to 2017. Researchers, civil society groups and government agency stuff could use such comprehensive celebrity endorsement information as a tool to develop policy statements and position papers that call on the industry to use celebrity marketing only to promote healthy food and beverage products.

The nutritional profile evaluation for the food and beverage products and brands supported the hypothesis for RQ2, which found that two-thirds of the brands, endorsements and celebrities were associated with EDNP food and beverage products (e.g., snacks, candy, SSB and QSR foods) that did not align with the USDA’s Smart Snacks Standards. This is a concern because overconsumption of these products is a significant contributor to the obesity epidemic [3,4,48]. We found that celebrities in the US have also been selectively used to endorse healthy food and beverage products such as NCBs and water, or health promotion and marketing campaigns such as the Drink Up Campaign and the FNV Campaign. Some celebrities were associated with both healthy and EDNP product endorsements. When one celebrity is involved in multiple brand or product endorsements, the promotion effects of one brand may spread to others [49]. Celebrities who endorse healthy nutritional profile products may serve as indirect endorsers of unhealthy nutritional profile products, and vice versa. The results of this study provide evidence for government agencies or other empowered bodies to develop policies to restrict celebrity endorsements that promote EDNP products while encouraging the use of this marketing strategy to promote healthy dietary behaviors.

Statistical analyses of the associations between celebrity demographic profiles and food and beverage nutritional profiles partially supported the second hypothesis in that male and Millennial celebrities were more likely to endorse EDNP food and beverage products than female and Generation Z celebrities. The logistic regression model indicates that male celebrities were 1.5 times more likely than female celebrities to endorse EDNP products. Previous research found that men tend to trust celebrity endorsements more than women [50,51,52]. Men were also reported to be more susceptible to and targeted by EDNP food marketers compared with women [50,51,52]. This result suggests that celebrity endorsement may contribute to poorer diet quality for men than women.

Age was also a significant indicator suggesting that Millennials were seven times more likely to endorse EDNP food and beverage products than Generation Z celebrities. Previous research showed that US Millennials adults are targeted more by food marketing due to their spending power ($1.4 trillion annually) [53]. Millennials also spent the highest budget on EDNP products including prepared foods, sugar, sweets and pasta than older generations [54]. However, Generation Z celebrities only represented 1.8% in the database, and Millennial celebrities were not more likely to endorse EDNP products than celebrities of other generations. Therefore, the results were not strong enough to establish the association between Millennial celebrities involved in EDNP products and the dietary or purchase behaviors of the Millennial consumers. Additionally, no statistical significance was observed to support the hypotheses that Black celebrities and sports celebrities were more likely to endorse food and beverage products and brands did not align with the USDA’s Smart Snacks Standards.

Therefore, we were not able to conclude that the food and beverage companies have intentionally selected Millennial, Black, and sports celebrities to endorse more EDNP products than healthy counterparts, compared with celebrities of other demographic characteristics. However, results from the descriptive statistical analysis for RQ1 found that most Millennial (32.2%, *n* = 99), Black (33.3%, *n* = 69) and sports (31.4%, *n* = 96) celebrities were associated with endorsements for SSB brands and products, which may contribute to over consumption of SSB products among the targeted populations.

Current governmental policies and industry self-regulatory programs have failed to protect Americans from food and beverage marketing [55]. The Children’s Food and Beverage Advertising Initiative (CFBAI) is an industry self-regulatory program established in 2006 to create more responsible food and beverage marketing practices for children under the age of 12 years. Yet after a decade, the CFBAI has failed to extend its pledges to adolescents, aged 12–18 years, who are targeted by celebrity endorsements of unhealthy food and beverage products [55]. The federal Interagency Working Group’s voluntary nutrition principles were drafted in 2011 to guide the marketing practices of industry self-regulatory programs [56]. The principles expended the definition of child-directed marketing up to adolescents aged 17 years but were not approved by the US Congress [56]. The HER’s responsible food marketing report published in 2015 can be used to guide the industry self-regulatory programs to create a more healthful child-marketing landscape, which suggests that the CFBAI should raise the coverage of their marketing regulations to children aged 14 years and younger [55]. The National Restaurant Association and Healthy Dining’s Kids LiveWell Program encourages more than 42,000 US restaurant locations to offer at least one healthy children’s meal that aligns with the calorie, fat, saturated fat and sodium recommendations for children aged 12 years and younger [57]. The USDA’s Smart Snacks Standards are beneficial for a life-long healthy dietary behavior, but these standards are designed to apply up to high school students or those 18 years of age [13]. There are limited US marketing standards established for young adults aged 18–25 years, who are influenced by food marketing but often neglected as a target population for obesity prevention [58]. There is a need to harmonize several nutrition guidelines to develop a universal set of standards for food and beverage product marketing to children, adolescents and adults, respectively.

### 4.2. Study Strength and Limitations

The strengths for this study are the large sample size compared to those used in previous US studies [29,30] regarding celebrity endorsement of food and beverage products. Another strength is the comprehensive analysis of the celebrity data. There are several limitations to this study. First, the celebrity endorsement data included in this study was collected via a non-randomized, convenience sample strategy which limits the generalization of the research results. Second, we had no access to proprietary data for the time course and contract duration of celebrity endorsements of food and beverage brands or products. Therefore, we were unable to accurately identify trends for using celebrity endorsements in the US food and beverage marketplace. Finally, the nutrition information and product ingredients may change over time due to industry reformulation to reduce or remove calories, trans fats, sugar and sodium. Therefore, the nutrition information for food and beverage products may not accurately reflect the nutrition profiles for these products during the duration of celebrity endorsements.

### 4.3. Implications for Future Research

First, several conceptual models have been widely applied in marketing to explain the influence of celebrity endorsements on people’s decisions and behaviors. However, there is limited research that has applied theoretical or conceptual models to guide the design of studies of celebrity endorsement for food, beverage and restaurant products. For example, it is not known whether celebrities who experience public scandals covered by the media may reduce people’s trust, brand loyalty and willingness to purchase branded food and beverage products endorsed. Future research could use marketing theories or conceptual models to understand the relationships between celebrity endorsement for food, beverage and restaurant products and the diet-related behaviors among targeted populations.

Second, experimental research is needed to understand the influence of celebrity endorsement on people’s consumption of food and beverage products in the US context. Experimental research could be conducted to explore whether racially, ethnically, culturally diverse, and different sex and age groups in the US respond differently to food and beverage products endorsed by celebrities, and which demographic groups are influenced by this marketing practice. It is also important to understand how people think about celebrities who endorse both healthy and unhealthy food and beverage groups and products.

Third, review studies are needed to evaluate the adequacy of the current policies and actions that diverse stakeholders have taken to ensure celebrity endorsement is used to promote products that support healthy food environments.

Finally, there is a need to compile a database regularly and prospectively with more comprehensive and detailed information to evaluate celebrity endorsements used in the US food and beverage marketplace. The database should include the start and end date of the endorsement relationships, the media channels that susceptible populations were most exposed to these endorsements, and the expenditure of using celebrities to promote food and beverage products. This database could be used to explore the shifting trends for the use of celebrity endorsement along the time period and if this marketing strategy is taking an increasing share in food and beverage marketing. The database should be continuously updated to reflect the use of celebrity endorsement in promoting food and beverage products in the US marketplace.

## 5. Conclusions

This is the first study to comprehensively examine the nature and extent of celebrity endorsements of branded food and beverage products and branded health promotion and marketing campaigns in the US. We found that celebrity endorsement was associated with a large proportion of EDNP products in the US marketplace. In addition, we identified a small percentage of celebrities who also were involved in promoting healthy food and beverage brands. This evidence can be used by government agencies, industry and consumer advocacy organizations to inform future US policies and actions that limit using celebrity endorsement to promote EDNP food and beverage products while encouraging this marketing practice to be applied to promote healthy nutrient-profile products, as part of a broader effort to engage in responsible food and beverage marketing practices that promote healthy food environments.

## Figures and Tables

**Table 1:** Food and beverage categories in the celebrity database.

Food and Beverage Categories	FTC ^1^	HER ^2^ and USDA’s Smart Snacks Standards ^3^
Breakfast cereals	X	
Dairy products	X	
Snacks and candy	X	
Prepared foods and meals	X	
Quick-service restaurant (QSR) foods	X	
Fruits and vegetables	X	
Sugar-sweetened beverages (SSBs)		X
Low-calorie beverages (LCBs)		X
No-calorie beverages (NCBs)		X
Water		X

^1^ Federal Trade Commission [28]; ^2^ Healthy Eating Research [42]; ^3^ US Department of Agriculture’s Smart Snacks Standards 2014–2015 [13].

**Table 2:** USDA’s Smart Snacks Standards 2014–2015 [13].

Nutrient	Snack	Entrée
Calories	200 calories or less	350 calories or less
Sodium	200 mg or less	480 mg or less
Total Fat	35% of calories or less	35% of calories or less
Saturated Fat	Less than 10% of calories	Less than 10% of calories
*Trans* Fat	0 g	0 g
Total Sugar	35% by weight or less	35% by weight or less

**Table 3:** Demographic profiles of celebrities (*n* = 542) who endorsed food and beverage products or brands in the US between 1990 and 2017.

Demographic Profile	Celebrities, *n* (%)
Profession category	
Sports/athlete	202 (37.3)
Entertainment	320 (59.0)
Other (e.g., chefs, political figures)	20 (3.7)
Age ^1^	
Generation Z (17–22 years)	10 (1.8)
Millennials (23–36 years)	211 (38.9)
Generation X (37–51 years)	215 (39.7)
Baby Boomers (52–66 years)	76 (14.0)
Silent Generation (67+ years)	30 (5.5)
Sex	
Male	332 (61.3)
Female	210 (38.7)
Race/ethnicity	
Black	138 (25.5)
White	338 (62.4)
Latino(a)	32 (5.9)
Asian	14 (2.6)
Multi-racial	20 (3.7)

^1^ Celebrity age was calculated according to the marketing age segment classification in 2017 [41].

**Table 4:** Associations between celebrity demographic profiles and food and beverage category endorsements (*n* = 732).

Celebrity Demographic Profiles		Food and Beverage Categories, *n* (%)										Total
		Breakfast Cereal	Dairy Products	Fruits and Vegetables	LCBs	NCBs	Prepared Foods	QSR Foods	Snacks and Candy	SSBs	Water	
Race/ethnicity	Black	6 (2.9)	33 (15.9)	33 (15.9)	4 (1.9)	3 (1.4)	6 (2.9)	26 (12.6)	19 (9.2)	69 (33.3)	8 (3.9)	207 (100)
	White	8 (1.9)	174 (40.9)	38 (8.9)	14 (3.3)	8 (1.9)	8 (1.9)	32 (7.5)	25 (5.9)	105 (24.7)	13 (3.1)	425 (100)
	Latino(a)	0 (0.0)	15 (33.3)	3 (6.7)	3 (6.7)	3 (6.7)	0 (0)	6 (13.3)	2 (4.4)	12 (26.7)	1 (2.2)	45 (100)
	Asian	0 (0.0)	4 (21.1)	1 (5.3)	1 (5.3)	2 (10.5)	2 (10.5)	3 (15.8)	3 (15.8)	3 (15.8)	0 (0)	19 (100)
	Multi-racial	1 (2.8)	5 (13.9)	8 (22.2)	4 (11.1)	1 (2.8)	1 (2.8)	4 (11.1)	2 (5.6)	7 (19.4)	3 (8.3)	36 (100)
Sex	Male	11 (2.4)	120 (26.3)	56 (12.3)	14 (3.1)	8 (1.8)	12 (2.6)	48 (10.5)	30 (6.6)	149 (32.6)	9 (2.0)	457 (100)
	Female	4 (1.5)	111 (40.4)	27 (9.8)	12 (4.4)	9 (3.3)	5 (1.8)	23 (8.4)	21 (7.6)	47 (17.1)	16 (5.8)	275 (100)
Profession category	Athlete	12 (3.9)	59 (19.3)	52 (17.0)	8 (2.6)	6 (2.0)	5 (1.6)	41 (13.4)	22 (7.2)	96 (31.4)	5 (1.6)	306 (100)
	Entertainer	3 (0.7)	169 (41.9)	27 (6.7)	18 (4.5)	10 (2.5)	6 (1.5)	25 (6.2)	28 (6.9)	100 (24.8)	17 (4.2)	403 (100)
	Other	0 (0.0)	3 (13.0)	4 (17.4)	0 (0.0)	1 (4.3)	6 (26.1)	5 (21.7)	1 (4.3)	0 (0.0)	3 (13.0)	23 (100)
Age	Generation Z (17–22 years)	2 (16.7)	2 (16.7)	4 (33.3)	2 (16.7)	0 (0.0)	0 (0.0)	1 (8.3)	0 (0.0)	0 (0.0)	1 (8.3)	12 (100)
	Millennials (23–36 years)	6 (2.0)	58 (18.9)	54 (17.6)	9 (2.9)	10 (3.3)	5 (1.6)	28 (9.1)	31 (10.1)	99 (32.2)	7 (2.3)	307 (100)
	Generation X (37–51 years)	6 (2.0)	91 (30.8)	21 (7.1)	11 (3.7)	4 (1.4)	8 (2.7)	36 (12.2)	17 (5.8)	88 (29.8)	13 (4.4)	295 (100)
	Baby Boomers (52–66 year)	1 (1.2)	55 (67.1)	4 (4.9)	3 (3.7)	3 (3.7)	4 (4.9)	4 (4.9)	1 (1.2)	5 (6.1)	2 (2.4)	82 (100)
	Silent Generation (67+ years)	0 (0.0)	25 (69.4)	0 (0.0)	1 (2.8)	0 (0.0)	0 (0.0)	2 (5.6)	2 (5.6)	4 (11.1)	2 (5.6)	36 (100)

**Table 5:** Alignments with the USDA’s Smart Snacks Standards [13] for the food and beverage endorsements (*n* = 510), brands (*n* = 119) and celebrities (*n* = 356).

Alignments with the Smart Snacks Standards	*n*	Percentage	*p*-Value
Endorsements for products or brands aligned	172	33.7	<0.001 *
Endorsements for products or brands not aligned	338	66.3	
Endorsed products or brands aligned	39	32.8	<0.001 *
Endorsed products or brands not aligned	80	67.2	
Celebrities only associated with endorsements aligned	119	33.4	<0.001 *
Celebrities associated with endorsements not aligned	237	66.6	

* Statistically significant.

**Table 6:** Associations between celebrity demographic profiles and the nutritional profiles of food and beverage product or brand endorsements (*n* = 510).

Celebrity Demographic Profiles		Alignments with the USDA’s Smart Snacks Standards [13]								
		Aligned			Not Aligned			χ^2^	Logistic Regression	
		Count	Column %	Row %	Count	Column %	Row %	*p*-Value	Odds Ratio	*p*-Value
Profession category	Sports/athlete	86	50.0	34.3	165	48.8	65.7	0.774		
	Entertainer	78	45.3	32.6	161	47.6	67.4		1.169	0.471
	Other	8	4.7	40.0	12	3.6	60.0		1.146	0.970
Sex	Male	104	60.5	30.3	239	70.7	69.7	0.020 *	1.546	0.041 *
	Female	68	39.5	40.7	99	29.3	59.3			
Age	Millennial (23–36 years)	91	52.9	35.5	165	48.8	64.5	0.002 *		
	Generation Z (17–22 years)	8	4.7	80.0	2	0.6	20.0		7.19	0.015 *
	Generation X (37–51 years)	56	32.6	27.3	149	44.1	72.7		0.717	0.126
	Baby Boomer (52–66 years)	14	8.1	50.0	14	4.1	50.0		2.096	0.088
	Silent Generation (67+ years)	3	1.7	27.3	8	2.4	72.7		0.764	0.704
Race/ethnicity	Black	56	32.6	32.6	119	35.2	68.0	0.184		
	White	83	48.3	32.5	172	50.9	67.5		1.021	0.924
	Latino(a)	10	5.8	30.3	23	6.8	69.7		0.918	0.842
	Asian	6	3.5	40.0	9	2.7	60.0		1.827	0.292
	Multi-racial	17	9.9	53.1	15	4.4	46.9		2.349	0.033 *

* Statistically significant. Notes: The column percentages represent the proportions of celebrities of different demographic categories involved with healthy or energy-dense and nutrient-poor (EDNP) product endorsements. The row percentages indicate the proportion of healthy or EDNP product endorsements associated with celebrities of each demographic characteristic. The first variable in each demographic group was set up as the reference variables (i.e., endorsements that were associated with sports, males, Millennials and Black celebrities) in the logistic regression model. The odds ratio quantifies how likely are the celebrity endorsements that are associated with products or brands that have nutritional profiles that are aligned with the Smart Snacks Standards compared with the reference variables.
